# The syllabic bridge: the first step in learning spelling-to-sound correspondences*

**DOI:** 10.1017/S0305000913000305

**Published:** 2013-09-16

**Authors:** NADEGE DOIGNON-CAMUS, DANIEL ZAGAR

**Affiliations:** Université de Strasbourg, France; Université de Lorraine, Nancy, France

## Abstract

It is widely agreed that learning to read starts with the establishment of letter-to-phoneme correspondences. However, it is also widely agreed that prereaders do not have access to phoneme units. Here we show that the building of associations between letters and syllables, which we call the ‘syllabic bridge’, might be a faster and more direct way of learning spelling-to-sound correspondences in French. After a few minutes of exposure, prereaders are able to learn the statistical properties of letter co-occurrences. Statistical learning is boosted by explicit instructions about the associations between letter clusters and syllables. Building the syllabic bridge from available phonological syllables and frequent letter clusters may therefore be the first step in learning to read.

## INTRODUCTION

The first steps in becoming literate require discovering the principles of phonological recoding and the acquisition of correspondences between orthographic patterns and speech sounds. Using and applying spelling-to-sound correspondences in decoding letter strings allow children to build the direct access to the mental lexicon (Jorm & Share, 1983; Share, 1995, 1999). The most common hypothesis in the literature is that children begin learning to read by learning letter-to-phoneme correspondences (Frith, 1985; Harm & Seidenberg, 1999; Seidenberg & McClelland, 1989). Therefore, children must be able to recognize individual letters, to isolate and compare phoneme units, and to map letters onto phonemes. However, this hypothesis presents a major problem, referred to as the ‘availability problem’, acknowledged by Ziegler and Goswami (2005) in their psycholinguistic grain size theory: not all phonological units are consciously accessible prior to reading acquisition (see Anthony & Francis, 2005, for a review). Studies of phonological awareness show that prereaders do not have access to phoneme units (Demont & Gombert, 1996; Liberman, Shankweiler, Fisher & Carter, 1974) suggesting that a lack of phonemic awareness leads to difficulties in mapping letters with phonemes at the first steps of reading acquisition.

Alternative assumptions to the first step of letter-to-phoneme acquisition have been explored. Based on the fact that rime awareness precedes phonemic awareness in children (Treiman & Zukowski, 1991, 1996), Treiman (1992) proposed that, in English, reading instruction that begins with onset–rime units may be more successful than instruction that begins at the phoneme level (see also Treiman, Mullennix, Bijeljac-Babic & Richmond-Welty, 1995). Similarly, Goswami and Bryant (1990; Bryant & Goswami, 1987; Goswami, 1999) suggested that children map letters with onsets and rimes, leading them to use analogies about the pronunciation of new words, right from the start of reading acquisition.

In French, several arguments raise the possibility of first spelling-to-sound correspondence acquisition based on syllabic units. Since the seminal works of Liberman *et al.* (1974), there is a large bulk of evidence showing that phonological awareness follows a large-to-small developmental sequence (Cassady, Smith & Putman, 2008; Treiman & Zukowski, 1996; Ziegler & Goswami, 2005). However, cross-language differences have been found. Duncan, Colé, Seymour, and Magnan (2006) showed that French-speaking children exhibited greater accuracy and consistency in manipulating syllables than English-speaking children. Long before reading instruction, at four years of age, French children have access to syllable units. Based on the availability of syllable units before reading acquisition, we consider an alternative hypothesis of literacy. Rather than map available orthographic units (i.e., letters) with phonological units (i.e., phonemes), the alternative hypothesis is that prereaders map available phonological units (i.e., syllables) with orthographic units (i.e., letter groups). Our hypothesis, which we call the ‘syllabic bridge hypothesis’, suggests that prereaders could learn print-to-sound associations by syllable-size units (Doignon-Camus & Zagar, 2009). This alternative hypothesis relies on speech primacy and takes the available syllable units as starting points of spelling-to-sound connections. In this case, the building of associations between letter clusters and syllables would be a direct and rapid way of learning the first spelling-to-sound correspondences.

The syllabic bridge hypothesis relies on the phonological availability of syllables but also on their visual availability. At first sight, letter clusters that correspond to phonological syllables do not seem to be available orthographic units. The reason is that letter clusters that form syllables are completely embedded in printed words. However, several studies using the illusory conjunction paradigm have provided evidence for an automatic perceptive segmentation of letter strings into syllable units (Doignon & Zagar, 2005; Prinzmetal, Treiman & Rho, 1986; Rapp, 1992). The illusory conjunction paradigm consists in briefly presenting a letter string in two colors. Participants are instructed to detect a target letter and to report its color. In some trials, an incorrect combination of the color and the letter occurs. As these feature integration errors, called illusory conjunctions, are more likely to occur within a perceptual group than between perceptual groups, they are thought to reflect the perceptual groups automatically evoked during word perception. For instance, the word ANVIL is presented two times, either ANVil or ANvil (in which upper- and lower-case letters represent two different colors). Prinzmetal *et al.* (1986) observed that participants made more illusory conjunctions that preserved the syllable boundary (e.g., for ANVil, reporting V to be the same color as IL) than illusory conjunctions that violated the syllable boundary (e.g., for ANvil, reporting V to be the same color as AN). Illusory conjunction studies clearly show that readers parse written words into letter groups that form phonological syllables in the first steps of word perception, at least in French (Doignon & Zagar, 2005) and in English (Prinzmetal *et al.*, 1986; Rapp, 1992). Similar results have been found in French beginning readers (Doignon & Zagar, 2006; Maïonchi-Pino, De Cara, Ecalle & Magnan, 2012a) and dyslexics (Fabre & Bedoin, 2003; Maïonchi-Pino, de Cara, Ecalle & Magnan, 2012b; but see Doignon-Camus, Seigneuric, Perrier, Sisti & Zagar, 2013). In addition to these illusory conjunction studies reporting that syllables are perceptive units, another set of studies clearly show that syllables are functional units of word processing in French beginning readers. The functional relevance of syllable units in lexical access has been demonstrated using the syllable frequency effect in a lexical decision task (Chetail & Mathey, 2009) or in a visual target detection task (Maïonchi-Pino, Magnan & Ecalle, 2010). In French beginning readers, syllables therefore appear to be perceptive units that play a role in visual word identification. The question that remains to be addressed is how readers can perceive such letter groups corresponding to syllable units when there is no clue indicating syllable boundaries in letter strings.

In fact, the correspondence between the salience of letter clusters and syllables in words is based on orthographic redundancy. Letters are not randomly distributed in written language, and, as Adams (1979) suggested, letter clusters that compose syllable units occur more frequently than letter clusters that straddle syllable boundaries. Syllable boundaries are therefore marked by a specific pattern of bigram frequencies, referred to as a ‘bigram trough’ (Seidenberg, 1987). For example, in the word ANVIL, the respective positional bigram frequencies around the syllable boundary are high–low–high for AN-NV-VI respectively. According to Seidenberg (1987), the lowest frequency bigram in this pattern allows syllable units in words to be isolated. Even if the bigram trough hypothesis has been disputed (Rapp, 1992), empirical data found with English (Seidenberg, 1987) and French expert readers (Doignon & Zagar, 2005) has confirmed the influence of orthographic redundancy on syllable perception. In both languages, illusory conjunctions were found to be affected by letter co-occurrence properties. Similar results have been reported with French beginning readers and have showed that children are sensitive to the frequency of letter cluster in letter strings, from the first year of reading acquisition (Doignon & Zagar, 2006). Children were able to use statistical orthographic properties to parse words into syllables, but the effects of such properties were not systematic (Maïonchi-Pino *et al.*, 2012a, 2012b).

In summary, there are two main arguments in favor of the syllabic bridge hypothesis. First, in the phonological bank of the syllabic bridge, syllables constitute accessible, mentally represented units. Second, in the orthographic bank, letter clusters corresponding to syllables are outlined using statistical properties. Investigations of the skills of beginning readers show that children are able to use statistical properties of orthographic redundancy to perceive syllable units in written words (Doignon & Zagar, 2006). Moreover, beginning readers use phonological grapho-syllabic processing of words (Chetail & Mathey, 2009; Maïonchi-Pino *et al.*, 2010), suggesting that connections are built between letter and syllable units. The aim of the present study was to test whether and how the syllable can be a unit of learning to read. Our hypothesis is that prereaders are able to build the first associations between orthographic and phonological patterns through syllable units. They should therefore be able to build the syllabic bridge. In the present study we investigated the ability of prereaders to learn letter co-occurrence properties, and the consequences of learning the letter-to-syllable correspondences.

First we examined the prerequisite of the syllabic bridge hypothesis, that is, sensitivity to orthographic redundancy. To learn statistical regularities of printed language, prereaders should use a statistical learning mechanism. A large proportion of studies that examined statistical learning were conducted using only oral language and showed that infants are able to encode statistical properties of oral language (Saffran, Aslin & Newport, 1996; Saffran, 2001; Seidenberg, 1997). In the visual modality, Kirkham, Slemmer, and Johnson (2002) showed that young infants were also able detect statistical regularities, providing evidence for the existence of a domain-general statistical learning mechanism. Recently, Cantlon, Pinel, Dehaene, and Pelphrey (2011) reported that four-year-old children develop sensitivity to printed symbols such as letters before learning to read. More importantly, the probability of letter sequences appears to modulate activation in the left occipito-temporal region at the site of the visual word form area (for English: Binder, Medler, Westbury, Liebenthal & Buchanan, 2006; for French: Vinckier, Dehaene, Jobert, Dubus, Sigman & Cohen, 2007), suggesting that this region becomes attuned to frequent letter groups in the course of learning to read. Such data are in agreement with the local combination detector model (Dehaene, Cohen, Sigman & Vinckier, 2005), in which a hierarchy of local combination detectors is associated with increasingly larger word fragments, suggesting that orthographic redundancy may be encoded by a group of neurons. With glyph material, Turk-Browne, Scholl, Chun, and Johnson (2008) showed that neural evidence of statistical learning appeared very quickly, as early as the second presentation of stimuli. Therefore, given that the ability to encode statistical orthographic properties arises as a result of learning from exposure to letter strings (Seidenberg & McClelland, 1989), we expect that after exposure to the same set of printed stimuli, prereaders would be able to learn statistical regularities and extract letter co-occurrence properties.

Second we explored the influence of visuo-phonological learning. Learning to read requires learning correspondences between orthographic and phonological segments of language. A learning session should therefore involve the matching of visual and phonological units presented simultaneously. Recently, a longitudinal training study showed that acquisition of letter-to-sound correspondences in non-reader kindergarten children resulted in emerging print sensitivity in cortical areas, mainly in the posterior visual word form area, suggesting that learning letter-to-sound correspondences play a central role (Brem *et al.*, 2010). One main point concerns the type of units used to learn the ortho-phonological correspondences. According to the syllabic bridge hypothesis, we expect that the availability of spoken syllables in prereaders facilitates mapping between letters and speech sounds. A French study on computer-assisted learning provided first evidence that audio-visual training based on the matching of phonological syllables and letter groups improves reading skills in poor readers at the beginning of learning to read (Ecalle, Magnan & Calmus, 2009).

**Fig. 1:**
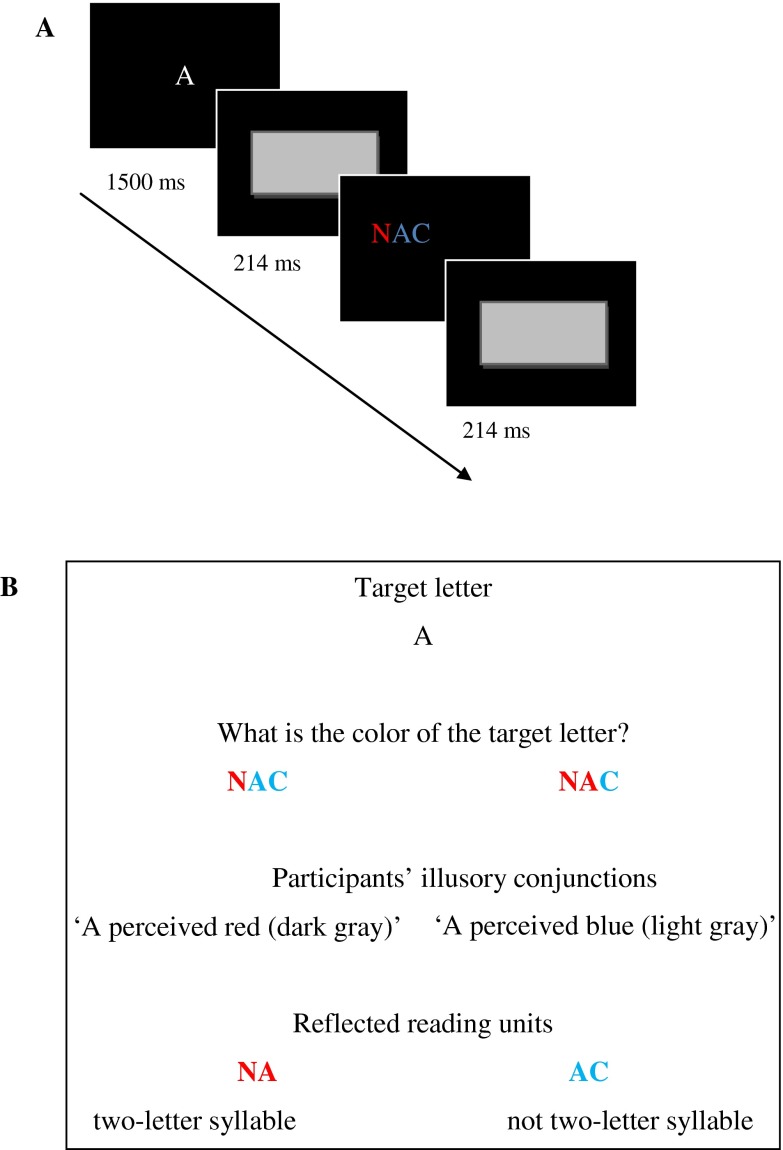
(Color online). Illustration of the illusory conjunction paradigm: A. Sequence of one trial in the task. B. The two types of illusory conjunctions are perception and non-perception of two-letter syllables.

To test the syllabic bridge hypothesis, and therefore both predictions, we presented prereaders with a learning program, assessed by two sessions of tests (pre- and post-tests) based on the illusory conjunction paradigm. Two experimental groups of prereaders studied four clusters of two letters that form a syllable (e.g., NA). Learning thus focused on syllable units, either with visual learning or visuo-phonological learning. In the visual learning group, the tasks required prereaders to pay attention to letter co-occurrences, enhancing their sensitivity to orthographic patterns (e.g., NA) and the associative linkage between their component letters. In order to promote visual learning of the letter group, it was important not just that prereaders passively looked at the letter group but that they paid careful attention to it. Statistical learning is indeed modulated by attention (Baker, Olson & Behrmann, 2004). Therefore, prereaders were asked to identify and name each unit of the letter group. In the visuo-phonological learning group, prereaders were asked to learn the correspondences between the letter clusters and the pronunciation of the syllable. A third group consisted of a control group of prereaders who completed an arithmetic exercise. Before and after the learning session, all the prereaders performed an illusory conjunction task (Figure 1) on three-letter strings (e.g., NAC), the beginning of which contained the learned two-letter syllable (e.g., NA). Prereaders could produce two types of illusory conjunctions. In the first, they could report that the target letter A was the same color as the letter N, indicating that they perceived the two-letter syllable. In the second, they could report that the letter A was the same color as the letter C, indicating non-perception of the two-letter syllable. It is worth noting that participants did not need to be readers, as the task only requires participants to detect the color of a target letter. As noted above, illusory conjunctions reflect the units of analysis of a letter string. In the present study, the use of this paradigm allowed us to determine the mental representations and processes automatically evoked when prereaders are presented with letter strings, before and after brief exposure to letter strings. In other words, the pattern of illusory conjunctions can reveal the first mechanisms involved in letter string processing. The data analysis consists of comparing illusory conjunctions indicating the perception of the two-letter syllable and illusory conjunctions indicating non-perception. If prereader children statistically learn letter distribution, then those prereaders who benefited from the visual learning program would detect the learned two-letter syllable in the three-letter string. In contrast to the control group, they should produce more illusory conjunctions indicating perception of the two-letter syllable than illusory conjunctions indicating non-perception. Furthermore, if the syllable is the most efficient way to learn print-to-sound correspondences, then explicit learning of the syllable pronunciation corresponding to the letter cluster should boost the perception of the learned two-letter syllable in the three-letter string. Illusory conjunctions indicating perception of the two-letter syllable should be more numerous than illusory conjunctions indicating non-perception after the visuo-phonological program than after the visual program.

## METHOD

### Participants

One hundred and sixty-two kindergarten-aged children (89 boys and 73 girls; mean age, 5·7 years; range, 5 to 6·2 years) were recruited in nursery schools. All were monolingual native French-speakers with no reported hearing problem and with normal or corrected vision. Written consent to participate was obtained from the school supervisor and from the teachers and parents of the 162 children. A first letter knowledge test required children to identify the name of sixteen uppercase letters. The participants selected for this study knew most of the letters (mean of 14·16 letters correctly named), but none of them had been taught to read. In France, teaching reading begins with reading instructions at the first year of compulsory schooling (at about age six), and parents don't typically teach reading at home. Kindergarten-aged participants were therefore referred to as prereader children. They were randomized to one of three learning sessions: visual (*n*=55), visuo-phonological (*n*=55), and arithmetic (*n*=52).

### Procedure

The same letter groups (PI, MU, LO, NA) were used in the visual and visuo-phonological learning sessions. All had a CV structure, which is the most frequent syllable structure in French. In order to control children's prior knowledge of print, the letter groups were chosen according to the following criteria: (a) they constituted a pseudo-word; (b) their bigram frequency was low (mean 1598, range from 735 to 2646); and (c) their phonological syllable frequency was also low (mean 388, range from 102 to 521). Bigram and syllable frequencies were computed from Manulex (Peereman, Lété & Sprenger-Charolles, 2007). Each letter cluster was printed on a white, 10×8 cm card.

#### Visual learning session

The session was composed of three parts. In the first part, a letter naming task, the child had to name the first letter printed on a card. If the response was correct, the experimenter approved, repeated the name of the letter, and asked the child to name the second letter used on the card; if the response was incorrect, the experimenter provided the correct name of the letter and asked the child to repeat the name, after which the experimenter started the exercise again. In the second part of the visual learning session, the experimenter shuffled the cards, randomly drew one, and presented the child with the same letter naming task as above. This task was repeated at least three times, more if participants still made mistakes, until all responses were correct. In the last part, the experimenter presented the four cards to the child and asked him or her to point to the card on which the two letters were printed. This exercise was performed at least twice, more if participants still made mistakes, until all responses were correct. The experimenter never pronounced the syllable formed by the two letters.

#### Visuo-phonological learning session

The session was also composed of two parts. The first part consisted of three tasks. The first task consisted of the same letter naming task as in the visual learning session. The second task consisted of a reading task, in which the experimenter showed the child a card and pronounced the syllable. Children had to repeat the syllable. If the child's pronunciation was correct, the experimenter approved, repeated the pronunciation of the syllable, and proceeded to the second card; if the child's pronunciation was incorrect, the experimenter started the task again. The third task consisted of learning the association between the letter clusters and the syllable pronunciation. The child was shown one card and the experimenter taught the association between letters and the syllable (e.g., ‘N and A make /na/. Can you repeat that?’). When the child correctly repeated the complete sentence, the experimenter approved, repeated the sentence, and turned to the second card. When the child made a mistake repeating the sentence, the experimenter began the task again until the child repeated the sentence correctly. In the second part of the visuo-phonological learning session, children again performed two tasks, a reading and a naming task. In the reading task, the experimenter randomly selected a card and asked the child to read the letter string. If the child's pronunciation was correct, the experimenter approved; if it was incorrect, the experimenter pronounced the letter cluster and asked the child to repeat it. This was followed by the naming task, in which the child was asked the names of the two letters from the reading task. If the response was correct, the experimenter approved; if the response was incorrect, the experimenter provided the correct letter names and asked the child to repeat them. Once both tasks had been performed using one card, the experimenter randomly selected a second card. Each child participated at least twice in the second part if they gave correct responses, until all responses were correct. In the third and last part of the visuo-phonological learning session, the experimenter checked learning. The four cards were presented and the experimenter asked the child to point to the card corresponding to each two-letter syllable that had been pronounced (e.g., the /na/ card). This exercise was performed at least twice, or until all responses were correct. The child was then asked to point to the card on which, e.g., N and A were printed. This exercise was performed at least twice, or until all responses were correct.

#### Arithmetic learning session

Children had to count several sets of three to ten tokens in 5 minutes. This arithmetic learning session was for the control group.

#### Duration of learning sessions

Each visual learning session lasted from 2 to 15 min (average 6 min) whereas each visuo-phonological learning session lasted from 3 to 17 min (average 9 min). The duration of the learning session was statistically controlled in the data analysis by introducing duration as a covariate.

#### Illusory conjunction task

The same illusory conjunction task was performed before and after each learning session. The experiment used the methodology (Figure 1) introduced by Prinzmetal *et al.* (1986, experiments 3–5). Each trial began with the presentation of a white letter in the center of the monitor. After 1500 ms the target was replaced by a gray rectangle for 214 ms. A string of three upper-case letters was briefly presented in one of the four corners and then replaced by a rectangle. Each letter string appeared in two colors randomly chosen among blue (light gray in print), yellow, and red (dark gray in print). Each letter string was presented twice: either the first two letters were in one color and the last letter was in another color (e.g., NAC, in which the two-letter syllable was in the same color (color online)), or the first letter was in one color and the last two letters were in another color (e.g., NAC, in which the two-letter syllable was not the same color). The target letter was always the central letter. To avoid any task-specific strategy, we added fillers that did not contain any letter cluster used in the learning session and in which the target letter was not central. Each session began with a practice block of thirty trials followed by two blocks of twenty trials each (4 letter strings created from the 4 learned units, each presented twice, and 8 fillers).

The duration of stimulus exposure was adjusted for each participant to maintain an error rate of approximately 20% throughout the experiment (Prinzmetal *et al.*, 1986). The initial exposure was set to twenty-five refresh cycles (357 ms). The duration was adjusted every ten trials in decrements and increments of one refresh cycle (14·28 ms). The mean exposure duration was 24·8 refresh cycles (354 ms) in the pre-test session and 22·24 refresh cycles (318 ms) in the post-test session. The illusory conjunction task was performed using three-letter strings (e.g., PID, MUB, LOC, NAC); the beginning of each string consisted of the letter clusters used in the training sessions (PI, MU, LO, NA). All three-letter stimuli thus had a CVC structure and formed pseudo-words. All stimuli had a low-frequency trigram (mean 10, range from 0 to 88) and a low-frequency syllable (mean 2, range from 0 to 31).

## RESULTS

As the present study used the illusory conjunction task with prereader children for the first time, we first provide a general description of the data we collected. The signal detection theory (Green & Swets, 1966; McMillan & Creelman, 2005) provides a general framework to describe the decisions that prereaders made in the illusory conjunction task, by measuring the discrimination sensitivity threshold (i.e., the *d′* criterion) and the response bias (i.e., the β criterion). In the present study, the discrimination sensitivity threshold reflects the capacity of prereaders to detect the correct color of the target letter among a degree of background noise. Firstly, the criterion *d′* was superior to 0, indicating that prereaders did not perform the illusory conjunction task by chance. The task is therefore suitable for non-reader children. Second, the criterion *d′* was consistent between the two sessions and between the three groups (pre-test: *d′*=2·33 for the visual group, *d′*=2·29 for the visuo-phonological group, and *d′*=2·34 for the control group; post-test: *d′*=2·45 for the visual group, *d′*=2·32 for the visuo-phonological group, and *d′*=2·42 for the control group). The constancy of *d′* values between groups and sessions was expected as the proportion of errors was fixed (i.e., 20%) throughout the illusory conjunction experiment by adjusting the duration of stimulus exposure (Prinzmetal *et al.*, 1986).

**Fig. 2.**
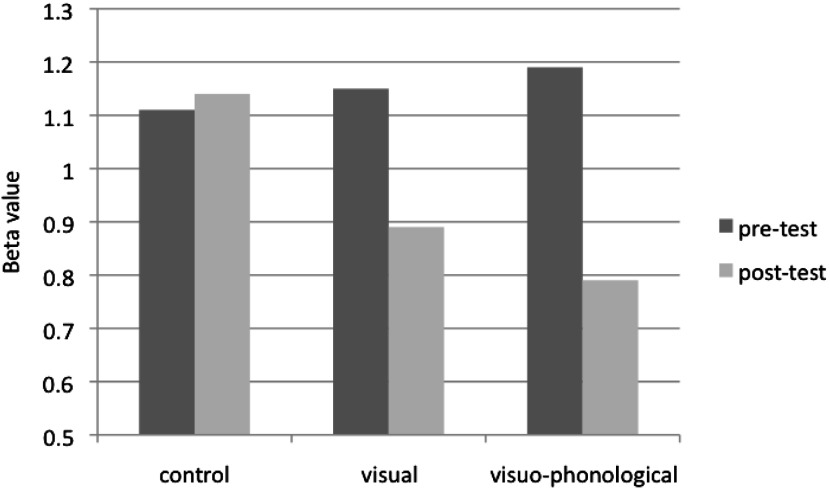
Beta values of the three learning groups and in the two test sessions.

In the present illusory conjunction task, the response bias β reflects the individual's general tendency to give a syllabic response (i.e., yes response) or a non-syllabic response (i.e., no response). Figure 2 presents the β values for the three groups. In the pretest, the β criterion was greater than 1 in the three groups (β=1·15 in the visual group, β=1·19 in the visuo-phonological group, and β=1·11 in the control group), suggesting a slight response bias toward the non-syllabic response. Such unexpected data mean that in the illusory conjunction pre-test, prereaders were slightly more likely to respond with the color of the last letter (i.e., the A in NAC is reported to be the same color as C), which may result from the sensitivity to the left-to-right reading direction. After the learning session, the β criterion was still greater than 1 (β=1·14) in the control group. However, the β criterion was less than 1 in the visual group (β=0·89) and in the visuo-phonological group (β=0·79), suggesting a response bias toward the syllabic response. In the illusory conjunction post-test, prereaders showed a bias towards responding with the first color (i.e., the A in NAC is reported to be the same color as N), which may result from the learning of the visual letter cluster and especially the visuo-phonological syllable unit.

The analysis of illusory conjunctions needs to observe the occurrences of illusory conjunctions and to compare the two types of conjunction errors (Prinzmetal *et al.*, 1986; Prinzmetal, Hoffman & Vest, 1991): those that indicate perception of the two-letter syllables and those that indicate non-perception of the two-letter syllables. The overall error rate was 20·15% in the pre-test and 16·02% in the post-test sessions. There were 5·03% and 3·27% off-screen errors (i.e., when participants reported a color that was not present in the stimulus), and 15·12% and 12·75% illusory conjunctions in the pre-test and post-test sessions respectively.

**Fig. 3.**
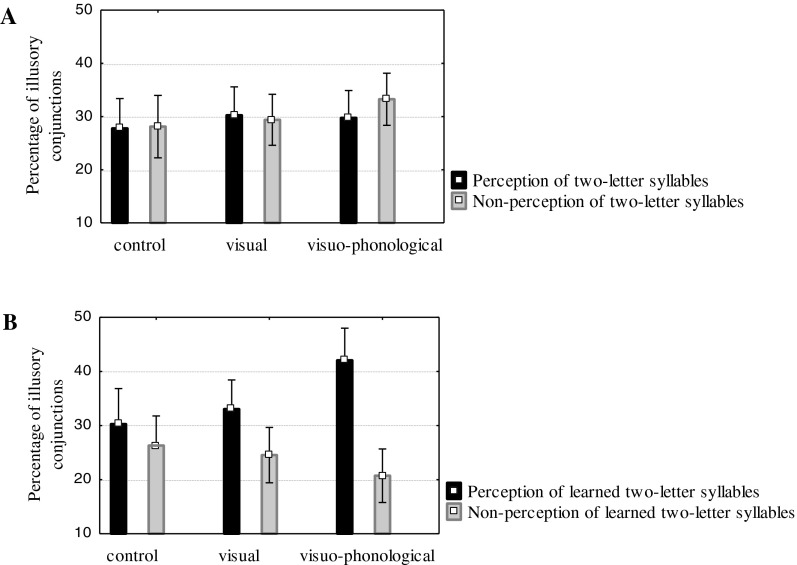
Percentage of illusory conjunctions in all experimental errors in the three groups: A. Pre-test. B. Post-test.

An analysis of variance was performed on the proportions of illusory conjunctions with respect to all the experimental errors, with stimulus display (indicating perception versus non-perception of the two-letter syllables) and session test (pre-test and post-test) within factors and a between-group factor (visual, visuo-phonological, and control). Mean percentages of illusory conjunctions are presented in Table 1. We observed a significant interaction between the two types of stimulus display, the two session tests, and the three groups (*F*(2, 159)=4·74, *p*=·009, η^*2*^*p*=·06). As shown in Figure 3, before the learning session there were no significant differences between illusory conjunctions resulting from the perception of syllable units (e.g., that the letter A was the same color as N in NAC) and illusory conjunctions resulting from the non-perception of syllable units (e.g., that the letter A was the same color as C in NAC), indicating that no reading unit was perceived within the letter strings (*F*<1 in the three groups). However, after the learning session, reading units emerged in both the visual (33·05% illusory conjunctions reflecting the perception of syllable units and 24·53% illusory conjunctions reflecting non-perception of syllable units; *F*(1, 159)=4·18, *p* =·04, η^*2*^*p*=·03) and the visuo-phonological (42·05% and 20·73%, respectively; *F1*(1, 159)=26·17, *p*<·0001, η^*2*^*p*=·14) groups but not in the control group (30·27% and 26·3%, respectively; *F*<1). Prereaders in the visual group were able to perceive, within the three-letter strings, the two-letter syllables they had repeatedly seen during the learning session. According to the statistical learning mechanism, these findings confirm that prereaders can encode statistical properties of letter distribution: the association between two letters was recognized after brief repeated attention to letter sequences (mean time, 6 min; range, 2 to 15 min). These results are in line with recent imaging data (Binder *et al.*, 2006; Turk-Browne *et al.*, 2008; Vinckier *et al.*, 2007) and the proposal of Dehaene *et al.* (2005), and highlight the power and the efficiency of the statistical learning mechanism in perceiving letter clusters in prereaders. More importantly, the learning of a letter cluster as a reading unit was strengthened when the letter sequence was explicitly associated with an available phonological representation, namely a syllable. The benefits of the visuo-phonological learning session were significantly greater than those of the visual-only learning session, as revealed by the significant interaction between stimulus display, session test, and the two groups (*F*(1, 108)=5·99, *p*=·01, η^*2*^*p*=·05). This result is new and clearly shows that syllable phonological units mapped to orthographic units improve learning of letter-to-sound correspondences. Learning the explicit syllabic bridge leads to a stronger perception of letter clusters corresponding to syllables than learning only orthographic statistical properties. It is worth noting that learning of the syllabic bridge took only a short time (mean time, 9 min; range, 3 to 17 min). In this very brief learning session, the availability of phonological syllables in prereader children boosted letter-to-sound mapping.

**Table 1.** Percentage of illusory conjunctions in the three learning sessions in the two test sessions

	Pre-test		Post-test	
	Preservations	Violations	Preservations	Violations
Visual learning	30·24	29·38	33·05	24·53
Visuo-phonological learning	29·74	33·26	42·05	20·73
Control group	27·77	28·1	30·27	26·3

As the ability to encode statistical properties arose as a result of exposure to letters, the last question is whether the difference between the visuo-phonological learning session and visual-only learning session merely reflected the fact that the former took longer (9 min vs. 6 min). An analysis of covariance was carried out with the duration of the session as a covariate. The interaction between stimulus display, session test, and the two groups was still significant (*F1*(1, 107)=4·9, *p*=·02, η^*2*^*p*=·04). In addition, we conducted the following post-hoc analysis. In each learning session, we formed two groups of prereaders based on the length of the learning session (i.e., short or long) and we observed the pattern of illusory conjunctions in the post-test. The results of Tukey's test showed no significant difference in the pattern of illusory conjunctions between shorter and longer learning sessions in either the visual or the visuo-phonological group. The benefit of the visuo-phonological learning session thus does not result from the longer duration (3 min). Finally, the fact that the pattern of illusory conjunctions was similar in both faster and slower learners also suggests that the building of the syllabic bridge does not vary as a function of the children's learning speed.

## DISCUSSION

The present findings are new and have important implications for literacy learning. The first result is that prereaders are able to learn statistical regularities of written language. This result provides evidence for a functional statistical learning mechanism (Kirkham *et al.*, 2002). Here we showed that this mechanism allowed children to process letter strings even though they have not yet developed literacy skills. Paying attention to the two letters presented simultaneously allowed the children to build an association between the two letter units. Therefore, as a result of brief exposure to letter strings, frequent cluster of letters were processed as a whole (Adams, 1979). According to the LCD model (Dehaene *et al.*, 2005), the neurophysiological basis for learning orthographic redundancy is the ventral visual word form: ‘as a result of exposure to print and adaptative learning processes, neural detectors have become dedicated to the recognition of frequent fragments’ of words (Vinckier *et al.*, 2007: 144). In children, the activation of the left ventral occipito-temporal region in response to written words increases with learning to read (Ben-Schachar, Dougherty, Deutsch & Wandell, 2011). Several studies have linked the neural activity of the visual word form area with the N170 component, thought to reflect expertise for print. In adult expert readers, larger N170 amplitudes have been found for word-like stimuli than for visual control stimuli such as symbol strings (Bentin, Mouchetant-Rostaing, Giard, Echallier & Pernier, 1999). Developmental studies reported that prereaders with high letter knowledge could produce similar results to expert readers, suggesting early print tuning (Maurer, Brem, Bucher & Brandeis, 2005). Therefore, taken together, previous findings and our current results show that non-reading children have the neural basis that enables them to rapidly develop letter cluster sensitivity.

The second result is that statistical learning is boosted by instructions about the phonological production corresponding to the letter cluster. The visuo-phonological learning session was very short (mean time 9 min) and focused on correspondences between letters and syllables. The effect of such learning was assessed in the illusory conjunction pattern, which showed that prereaders clearly perceived learned syllable units. This is evidence that the syllabic bridge has been built. Brem *et al.* (2010) similarly investigated the effects of learning spelling-to-sound correspondences on neural changes in non-reading children. Contrary to our study, learning was intensive (a period of 58·1 days; mean 3·6 h) and focused first on correspondences between individual letters and sounds, then between letter groups and sounds, and finally between printed words and pseudo-words and their pronunciation. After learning, ERP and fMRI assessment showed an initial sensitization to print, with activation of the occipito-temporal region and N1 effects. Based on our present findings, future studies should test whether a shorter learning focused on letter-to-syllable correspondences is effective in initiating print sensitivity in non-reading children. Our hypothesis is that building letter-to-syllable associations will be evidenced by neural changes, particularly by the emergence of print sensitivity of the visual word form system.

The phenomenon of the strengthening of implicit statistical learning with an explicit instruction about letter-to-syllable correspondences is consistent with the syllabic bridge hypothesis. As noted above, several developmental studies with beginning readers have reported that syllables are perceptual and functional units in visual word processing (Chetail & Mathey, 2009; Doignon & Zagar, 2006; Maïonchi-Pino *et al.*, 2010). Here we show that syllables are perceptual and functional units in the process of reading acquisition. The syllabic bridge hypothesis is the following: the first step of reading acquisition consists of mapping pre-existing phonological units (i.e., syllables) with orthographic units (i.e., letter groups). Our data showed that children without literacy abilities are able to build associations between letter and syllable units. This striking result showed that the bridge between the orthographic and phonological banks can be preferentially built through syllable units. Therefore, before learning letter-to-phoneme mapping, the syllabic bridge may be the first step in reading acquisition. Once the syllabic bridge is built, beginning readers would be more able to map correspondences between the smallest units of written and spoken language, between single letters and phonemes.

**Fig. 4.**
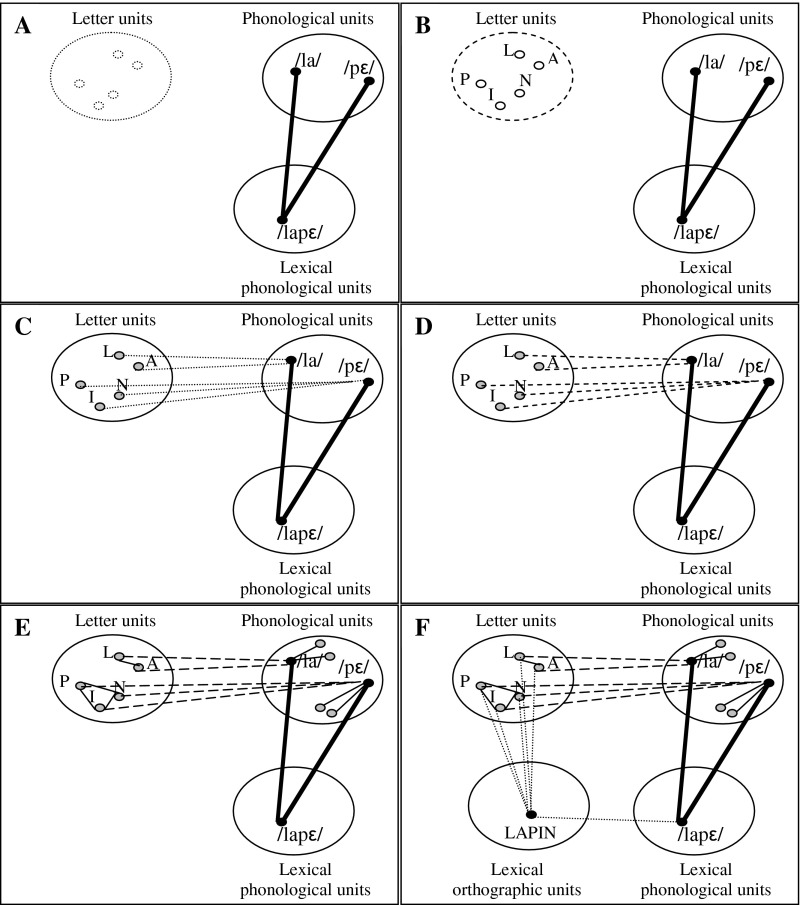
Developmental interactive model with syllables (DIAMS). notes: A. Linguistic system before learning to read, with lexical phonological representations. B. Construction of orthographic representations. C. Mapping letter clusters to available phonological syllables. D. Automation of letter-to-syllable mapping. E. Construction of phonemic representations and strengthening of inter-letter connections. F. Construction of lexical orthographic representations.

Recently, Grainger and Ziegler (2011) described a dual-route approach to orthographic processing in a developmental perspective and proposed that orthographic codes in skilled reading and in learning to read are based on the frequency of letter co-occurrence. Chunking frequent combinations of adjacent letters is thought to facilitate the mapping of letter cluster representation onto pre-existing phonological representations. Our data clearly showed that that prereaders are able to chunk letter clusters that occur repeatedly and that they are able to map them onto pre-existing phonological representations. More precisely, the pre-existing phonological representations that could be easily and rapidly mapped with letter clusters are actually syllable units. These data are in line with the framework of a developmental approach of learning to read (see Figure 4; Doignon-Camus & Zagar, 2009) in which syllables are the elementary phonological units represented before reading acquisition. According to this theoretical hypothesis, the first-ever connections between printed and spoken language are connections between letter groups and the available phonological syllables. Thereafter, the automation of mapping letter cluster to phonological syllables enables phoneme units to become accessible and to be the mirror of the letters. As Adams (1981) suggested, the connection of several letters to a phonological syllable also strengthens the probability to chunk letters, and therefore, in turn, develops sensitivity to orthographic redundancy. The key point of this theoretical proposal is that learning to read consists in mapping available phonological units with orthographic units.
